# Annual Meeting and Distribution of Grants

**Published:** 1912-12-28

**Authors:** 


### KING EDWARD'S HOSPITAL FUND FOR LONDON.

### Annual Meeting and Distribution of Grants.

A meeting of the Governors and General Council of King Edward's Hospital Fund for London, for the purpose of awarding grants to the hospitals, convalescent homes, and consumption sanatoria, was held on the 18th inst. at York House, St. James's Palace, the Speaker of the House of Commons being in the chair. [The Speaker's address at the meeting was fully reported in The Hospital last week.]

Those present were the Viscount Iveagh, the Duke of Norfolk, Lord Rothschild, Lord Revelstoke, Lord Farquhar, Lord Stamfordham, the President of the Hospital Sunday and Hospital Saturday Funds (the Right Hon. the Lord Mayor), the Chairman of the County Council (Lord Cheylesmore), the President of the Royal College of Physicians (Sir Thomas Barlow, Bart.), the Right Hon. Sir Savile Crossley, Bart. (Hon. Secretary), the Right Hon. Sir Edgar Speyer, Bart., Sir W. S. Church, Bart., Sir Henry Burdett, Sir Horace Marshall, Sir William J. Collins, Sir Cooper Perry, Sir John Craggs, Mr. J. Danvers Power, Mr. Frederick M. Fry, Dr. Edwin Freshfield, Mr. John G. Griffiths (Hon. Secretary), Mr. William Latham, K.C., Mr. Albert G. Sandeman.

Lord Rothschild (the Hon. Treasurer), in reporting that the amount received for general purposes by the Fund to December 16, after payment of expenses, was £117,338 10s. 3d., said that, for the first time for several years, the total of the accounts was not of the satisfactory character which could be desired. There was a deficit on the year; that deficit would be met out of accumulated surpluses not distributed in previous years. He did not think it would be proper to analyse the reasons why the present year had not been of quite the satisfactory nature that past years had been. This year the legacies had unfortunately been smaller. It was only to be hoped that in years to come the prosperity of this great Fund would not be impaired by future legislation making calls upon the public. It was only right to mention that Messrs. Barings, who held the funds on deposit, had allowed the Fund a most generous rate of interest.

### The Work of the League of Mercy.

Sir Henry Burdett, in making his annual statement on behalf of the League of Mercy, said that this had been a year of great anxiety, but it had called forth increased effort on the part of the League, and it was a matter of great satisfaction to be able to report that a larger sum had been raised this year than last, and he hoped that the grant to be made to the King's Fund by the League of Mercy would be at least equal to that of 1911. As to the future, the announcement by the Insurance Commissioners that morning would, he thought, help forward the League of Mercy; it meant that the poor, who were their immediate care, would need their help more than ever before. The members hoped next year to raise a still larger sum and to do an infinitely greater amount of good by being not the Levite, but the Good Samaritan in reference to the hospitals.

The actual sum raised by the League this year was £20,000. They had given altogether £182,000 through the King's Fund and contributions to hospitals in the home counties in the past twelve years. If a few friends would send to him as Treasurer of the League before the end of the year £1,000, the League would be in the proud position of having contributed £200,000 in twelve years for the sick poor of this country. If that additional £1,000 were forthcoming within the next few days, it would so inspirit their members that next year their collection would be larger than it had ever been in the history of the League.

### The Distribution Committee's Report.

Sir William Church (the Chairman of the Distribution Committee) read the report of that committee as follows :—

The Governors and General Council having again placed the sum of £151,000 in the hands of the committee with a view to distribution amongst the London hospitals, the committee beg to report as follows :—

1. The number of hospitals applying for grants is 104, as against 105 last year.

2. St. George's Hospital, which withdrew its application last year as a result of the decision of the Fund in March 1911 on the question of the amounts paid and allowed by the hospital to the medical school in respect of work done for the hospital, has applied again this year. From the accounts submitted on April 13, 1912, in support of this application it appeared that the payments in question were calculated on a method similar to that adopted on the occasion when the grant was withheld, and the attention of the hospital was called to this fact a fortnight later. To this communication no reply was received, and down to the time when the annual distribution of 1912 was approaching, no evidence had been addressed to the Fund by the hospital in support of its method of apportioning laboratory expenditure between hospital and school, and no information had been offered as to the grounds on which the hospital did not accept the previous decision of the Fund disapproving of that method. The committee, however, learned from statements in the press and in the annual report of the hospital that evidence on these points had been furnished in February to the Lord Mayor on behalf of the Hospital Sunday Fund, and on October 14, in view of their approaching meetings to consider grants to hospitals, they requested that this evidence might also be furnished to this Fund. It was not until November 20, however, that this evidence was received. It was at once referred to a sub-committee, but the necessity of comparing and verifying a considerable number of items in the various returns of the hospitals which have supplied information to the Fund and to St. George's Hospital has rendered it impossible for the sub-committee to complete their work; and, though an interim report indicating the chief points of difference has already been furnished, the committee are not yet able to report whether or not the fresh evidence affords any ground for modifying their previous conclusion. As a result, therefore, of the lapse of time before this information was received, the committee are not in position to make any recommendation upon the application of St. George's for a grant this year.

3. It was decided last year that the grants promised or set aside towards a general amalgamation of the five Throat, Nose, and Ear Hospitals should remain at the figure of £20,500 pending an agreement on a satisfactory scheme or schemes. A scheme submitted by three of the hospitals is at present under discussion. Except in this case, none of the grants made to special purposes in 1910 remain unapplied, and no question of renewal under the two years' rule arises.

4. As in past years several grants appear in the list of awards in aid of schemes of improvement or extension which have been submitted to the Fund in accordance with the resolution of the General Council to that effect.

5. The sum of £5,000 voted towards the removal of King's College Hospital to South London increases the total amount contributed by the Fund to this object to £48,000. The committee are informed that the new hospital will probably be opened in the autumn of 1913.

THE BRITISH LYING-IN HOSPITAL REMOVAL.

6. Some time ago the Fund was approached by the authorities of the British Lying-in Hospital, Endell Street, who desired to discuss the question of the future of the hospital in view of the changes in the character of its immediate neighbourhood. The Distribution Committee have now learned with satisfaction that the Charity Commissioners are prepared to consider favourably a scheme for an amalgamation between the British Lying-in Hospital and the Home for Mothers and Babies at Woolwich.

7. The reports of the visitors have again proved to be of the greatest value, and many of their observations have been brought to the notice of the hospital authorities. The committee have also communicated in certain cases private remarks arising out of their own examination of the circumstances of particular hospitals, including in more than one instance a suggestion that greater efforts should be made to obtain support from the general public.

8. The committee have also drawn the attention of the hospitals concerned to cases where it appears from the Fund's annual statistical report that the excess of cost above the average, in items more or less readily controllable from year to year, calls either for justification or for the exercise of greater economy.

THE DISTRIBUTION COMMITTEE'S AWARDS.

(The Distribution Committee desire to draw attention to the fact that the absence of a grant does not necessarily imply dissatisfaction.)

Sir Savile Crossley (Hon. Secretary) presented the list of awards as follows:—

Alexandra Hospital for Children.—£300.

Beckenham Cottage Hospital.—£25.

Belgrave Hospital.—£1,250; of which £500 to reduce building debt.

Blackheath and Charlton Hospital.—£150.

Bolingbroke Hospital.—£1,300.

British Lying-in Hospital.—£500.

Central London Ophthalmic Hospital.—£1,500; to rebuilding in accordance with the scheme submitted to the Fund.

Central London Throat and Ear Hospital.—Capital grants amounting to £20,500 have been set aside and promised for the purpose of a general amalgamation of the Throat, Nose, and Ear Hospitals. See last year's Report.

Charing Cross Hospital.—£4,000; of which £1,000 to redemption of mortgage.

Chelsea Hospital for Women.—£1,500; of which £1,000 to rebuilding in accordance with the scheme submitted to the Fund.

Cheyne Hospital for Sick and Incurable Children.—£100; in consideration of the fact that some curable cases are admitted.

City of London Hospital for Diseases of the Chest, Victoria Park.—£3,500.

City of London Lying-in Hospital.—£1,000; of which £500 to reduce building debt.

Clapham Maternity Hospital.—£200.

Dreadnought Hospital.—£4,500; of which £500 to reduce debt and £500 to renewal of floors.

East End Mothers' Lying-in Home.—£200.

East London Hospital for Children.—£2,250; of which £500 to reduce debt on recent improvements.

Eltham and Mottingham Cottage Hospital.—£100.

Finchley Cottage Hospital.—£25.

Florence Nightingale Hospital for Gentlewomen.—£200.

French Hospital.—£200. Friedenheim Hospital.—£50.

General Lying-in Hospital.—£350. German Hospital.—£200.

Gordon Hospital for Fistula, etc.—£25.

Great Northern Central Hospital.—£4,000; of which £1,000 to reduce debt.

Grosvenor Hospital for Women.—£350.

Guy's Hospital.—£7,500; of which £2,500 to building improvements in accordance with the scheme submitted to the Fund.

Hampstead General Hospital.—£3,250; of which £1,650 on account of maintenance and rent, as agreed upon amalgamation with the North-West London Hospital, and £1,500 to extinguish building debt.

Home and Infirmary for Sick Children.—£250.

Home for Mothers and Babies, Woolwich.—£100.

Hospital and Home for Incurable Children, Hampstead.—£50; in consideration of the fact that some curable cases are admitted.

Hospital for Consumption, Brompton (including Sanatorium at Frimley).—£6,000; of which £1,500 to reduce debt.

Hospital for Diseases of the Throat.—Capital grants amounting to £20,500 have been set aside and promised for the purpose of a general amalgamation of the Throat, Nose, and Ear Hospitals. See last year's Report.

Hospital for Epilepsy and Paralysis.—£1,500; of which £1,000 to extension in accordance with the scheme submitted to the Fund.

Hospital for Sick Children.—£2,500.

Hospital for Women, Soho Square.—£2,000; of which £1,000 to reduction of mortgage on No. 4 Frith Street.

Hospital of St. John and St. Elizabeth.—£350.

Infants Hospital.—£250. Italian Hospital.—£400.

Kensington and Fulham General Hospital.—£25.

Kensington Dispensary and Children's Hospital.—£25.

King Edward Memorial Hospital (Ealing).—£600; of which £275 to extinguish debt on the rebuilding of the hospital.

King's College Hospital.—£7,000; of which £5,000 to Removal Fund.

Leyton, Walthamstow, and Wanstead Children's and General Hospital.—£300.

London Fever Hospital.—£50.

London Homœopathic Hospital.—£400.

London Hospital.—£12,500; of which £2,500 to reduce debt on recent improvements.

London Lock Hospital.—£4,000; of which £500 to the Harrow Road Hospital, and £3,500 to the rebuilding of the Dean Street Hospital in accordance with the scheme submitted to the Fund.

London Temperance Hospital.—£1,500.

London Throat Hospital.—Capital grants amounting to £20,500 have been set aside and promised for the purpose of a general amalgamation of the Throat, Nose, and Ear Hospitals. See last year's Report.

Maternity Charity and District Nurses' Home, Plaistow.—£150.

Medical Mission Hospital, Balaam Street, Plaistow.—£350.

Memorial Hospital, Mildmay Park.—£50.

Metropolitan Hospital.—£3,500.

Middlesex Hospital.—£4,500; of which £500 to recent improvements in accordance with the scheme submitted to the Fund.

Middlesex Hospital Cancer Charity.—£250.

Mildmay Mission Hospital.—£400.

Miller General Hospital for South-East London.—£2,750; of which £2,000 to extension in accordance with the scheme submitted to the Fund.

Mount Vernon Hospital for Consumption (including Sanatorium at Northwood).—£3,000.

National Dental Hospital.—£250.

National Hospital for Diseases of the Heart.—

National Hospital for the Paralysed and Epileptic.—£2,500; of which £500 to reduce debt.

Nelson Hospital (late South Wimbledon, etc.).—£1,000; of which £750 to rebuilding in accordance with the scheme submitted to the Fund.
New Hospital for Women.—£750.
Norwood Cottage Hospital.—£50.
Paddington Green Children's Hospital.—£800; of which £200 to the completed reconstruction of out-patient department in accordance with the scheme submitted to the Fund.
Passmore Edwards Acton Cottage Hospital.—£75.
Passmore Edwards East Ham Hospital.—£50.
Passmore Edwards Hospital for Willesden.—£25.
Passmore Edwards Cottage Hospital, Wood Green.—£25.
Poplar Hospital.—£600; of which £200 to improvements in accordance with the scheme submitted to the Fund.
Prince of Wales's General Hospital.—£4,000; of which £1,500 to reduce debt.
Queen Charlotte's Lying-in Hospital.—£1,000; of which £250 to improvements in accordance with the scheme submitted to the Fund.
Queen's Hospital for Children.—£3,250; of which £1,250 to reduce building debt.
Royal Dental Hospital of London.—£750; of which £500 to reduce building debt.
Royal Ear Hospital.—Capital grants amounting to £20,500 have been set aside and promised for the purpose of a general amalgamation of the Throat, Nose, and Ear Hospitals. See last year's Report.
Royal Eye Hospital.—£750.
Royal Free Hospital.—£7,000; of which £5,000 to new out-patient department in accordance with the scheme submitted to the Fund.
Royal Hospital for Diseases of the Chest, City Road.—£1,500; of which £250 to reduce debt.
Royal Hospital, Richmond.—£200.
Royal London Ophthalmic Hospital.—£2,250.
Royal National Orthopædic Hospital.—£4,000; of which £2,000 to reduce building debt.
Royal Waterloo Hospital for Children and Women.—£1,500.
Royal Westminster Ophthalmic Hospital.—£100.
St. George's Hospital.—See paragraph 2 of Report.
St. John's Hospital for Diseases of the Skin.—£100.
St. John's Hospital, Lewisham.—£350.
St. Luke's House.—£50. St. Mark's Hospital.—£400.
St. Mary's Hospital.—£5,500; of which £1,000 to reconstruction of casualty department in accordance with the scheme submitted to the Fund.
St. Mary's Hospital for Women and Children, Plaistow.—£750.
St. Monica's Home Hospital.—£75.
St. Peter's Hospital for Stone.—£200.
St. Saviour's Hospital, Osnaburgh Street.—£150.
Samaritan Free Hospital.—£1,500; of which £500 to new Nurses' Home in accordance with the scheme submitted to the Fund.
Santa Claus Home.—£25.
University College Hospital.—£4,500.
Victoria Hospital for Children.—£1,000.
West End Hospital for Diseases of the Nervous System.—£350.
Western Ophthalmic Hospital.—£350; to reduction of debt on rebuilding of out-patient department.
West Ham and Eastern General Hospital.—£2,500.
West London Hospital.—£4,575; of which £1,075 to reduce debt and £500 to new operating theatre in accordance with the scheme submitted to the Fund.
Westminster Hospital.—£3,000.
Wimbledon Cottage Hospital.—£150; of which £100 to rebuilding in accordance with the scheme submitted to the Fund.

**Total grants to hospitals for the year 1912, £151,000**

The Convalescent Homes Committee.

Mr. William Latham, K.C. (the Chairman of the Convalescent Homes Committee), read the report of that committee as follows:—

1. The Governors and General Council have this year again placed the sum of £6,500 in the hands of the committee with a view to distribution amongst convalescent homes and consumption sanatoria.

2. The number of applications eligible for consideration amounted to 44 from convalescent homes and 10 from consumption sanatoria, as against 43 and 7 respectively last year.

3. In dealing with applications from convalescent homes the committee have adhered to their original policy of giving special consideration to the convalescent homes closely connected with London hospitals.

4. The committee are glad to report that the scheme for placing beds in country consumption sanatoria at the disposal of patients in London hospitals continues to prove satisfactory. The amount of accommodation offered has increased considerably. But in view of the sum available for distribution and of the prevailing uncertainty as to the effect of the arrangements for sanatorium benefit under the National Insurance Act, the committee did not feel justified in adding materially this year to the number of beds taken by the Fund. For the present, therefore, they have secured thirty-nine beds at five sanatoria as against thirty-seven beds at four sanatoria last year. The accommodation is situated as follows:—

Two beds for children at the Maitland Sanatorium, Peppard, Oxon.

Seven beds at the Sanatorium of the National Association for the Establishment and Maintenance of Sanatoria for Workers, Benenden, Kent.

Eight beds at the Kelling Sanatorium, Holt, Norfolk.

Ten beds at the Devon and Cornwall Sanatorium, South Brent, Devon.

Twelve beds at the Northampton Sanatorium, Creaton, Northants.

5. With regard to the allocation of these beds to hospitals in London the committee have followed the same principle as that adopted last year, viz.: that preference should be given (in order of relative size) to the two consumption hospitals which have no country branches and that the rest of the beds should be divided between the two largest of the general hospitals which apply to the Fund.

They accordingly recommend the following allocation:—

Eighteen beds to the City of London Hospital for Diseases of the Chest. Nine beds to the Royal Hospital for Diseases of the Chest.

Six beds to the London Hospital.

Six beds to Guy's Hospital.

6. The committee desire to express their thanks to the visitors who inspected the sanatoria applying for grants, and who thus furnished the committee with invaluable evidence as to their eligibility.

7. The grants recommended to the various institutions are appended.

The Convalescent Homes Committee's Awards.

(The Convalescent Homes Committee desire to draw attention to the fact that the absence of a grant does not necessarily imply dissatisfaction.)

Mr. John G. Griffiths (Hon. Secretary) presented the list of awards as follows:—

A.—Consumption Sanatoria.

Children's Sanatorium, Holt, Norfolk.—£50. To equipment.
Church Army Sanatorium (Children), Fleet, Hants.—
Daneswood Sanatorium (Jewish), Woburn Sands.—£50.
Devon and Cornwall Sanatorium, Didworthy, South Brent.—£875. In consideration of the reservation of ten beds during 1913 for the use of certain London hospitals. (See paragraphs 4 and 5 of Report.)
Eversfield Chest Hospital, St. Leonards.—£50.
Fairlight Sanatorium, Hastings.—£25.
Kelling Sanatorium, Holt, Norfolk.—£700. In consideration of the reservation of eight beds during 1913 for the use of certain London hospitals. (See paragraphs 4 and 5 of Report.)

Maitland Sanatorium, Peppard, Oxon.—£125. In consideration of the reservation of two children's beds during 1913 for the use of certain London hospitals. (See paragraphs 4 and 5 of Report.)

National Association for the Establishment and Maintenance of Sanatoria for Workers, Benenden, Kent.—£625. In consideration of the reservation of seven beds during 1913 for the use of certain London hospitals. (See paragraphs 4 and 5 of Report.)

Northampton Sanatorium, Creaton, Northants.—£1,050. In consideration of the reservation of twelve beds during 1913 for the use of certain London hospitals. (See paragraphs 4 and 5 of Report.)

Total ... ... ... ... £3,550.

B.—Convalescent Homes.

(The names of convalescent homes receiving no grant are not published.)

Alexandra Hospital for Children, Painswick.—£25.

All Saints' Convalescent Home for Children, Highgate.—£25.

Baldwin Brown Home for Convalescent Poor, Herne Bay.—£25.

Beau Site Convalescent Home, Hastings.—£25.

Brentwood Convalescent Home for London Children, Brentwood.—£25.

Catherine Gladstone Free Convalescent Home for the Poor, Mitcham.—£50.

Charing Cross Hospital, Limpsfield.—£125. With a view to encouraging the admission without payment of patients direct from the hospital.

Chelsea Hospital for Women, St. Leonards.—£50. With a view to encouraging the admission without payment of patients direct from the hospital.

Children's Cottage Hospital, Cold Ash, Newbury.—£25. In consideration of convalescent cases.

Children's Home Hospital, Barnet.—£25. In consideration of convalescent cases.

Convalescent Police Seaside Home, Hove.—£25.

Deptford Medical Mission Convalescent Home, Bexhill.—£25.

East London Hospital for Children, Bognor.—£225.

Erskine House, Hampstead Heath.—£50; of which £25 to reduce debt.

Friendly Societies' Convalescent Homes, Dover and Herne Bay.—£50.

Great Northern Central Hospital, Clacton-on-Sea.—£125.

Home and Infirmary for Sick Children (Sydenham), Broadstairs.—£50.

Hospital for Sick Children (Great Ormond Street), Highgate.—£100.

Jewish (Children) Convalescent Home, Brighton.—£25.

King's College Hospital, Hemel Hempstead.—£100.

London Hospital Convalescent Home, Tankerton.—£75.

Mary Wardell Convalescent Home for Scarlet Fever, Stanmore.—£25.

Metropolitan Convalescent Institution (Children), Broadstairs.—£50.

Metropolitan Convalescent Institution, Walton.—£150.

Middlesex Hospital, Clacton.—£400.

Mildmay Mission Hospital, Hendon.—£75.

Mrs. Kitto's Free Convalescent Home, Reigate.—£75.

National Hospital for the Paralysed, Finchley.—£100. With a view to facilitating the admission of patients without payment.

Paddington Green Children's Hospital, Slough.—£100.

Poplar Hospital, Walton-on-Naze.—£50.

Queen Charlotte's Lying-in Hospital, Kilburn.—£50.

Queen's Hospital for Children, Bexhill.—£100.

St. Andrew's Convalescent Home, Folkestone.—£50.

St. Andrew's Convalescent Hospital, Clewer.—£25.

St. Mary's Home for Children, Broadstairs.—£75. In consideration of convalescent cases.

St. Michael's Home for Men and Women, Westgate-on-Sea.—£25.

Sunshine Home of Recovery, Hurstpierpoint.—£50; of which £25 to reduce debt.

Surgical Home for Boys, Banstead.—£25.

Victoria Home for Invalid Children, Margate.—£25. In consideration of convalescent cases.

Victoria Hospital for Children (Chelsea), Broadstairs.—£225.

Winifred House Invalid Children's Convalescent Hospital Home, Tollington Park.—£25.

Total ... ... ... ... ... £2,950.

Grants to consumption sanatoria ... ... ... £3,550
Grants to convalescent homes ... ... ... ... 2,950

**Total grants recommended ... ... £6,50**

The Speaker of the House of Commons then moved the adoption of the reports and awards. (This speech was published in full in The Hospital of December 21, pp. 325-6.)

Lord Stamfordham having seconded the motion, the adoption of the reports was then put and carried unanimously.

Lord Iveagh then said: Mr. Speaker, my Lords and Gentlemen,—I beg to move that a vote of humble thanks be tendered to His Majesty for his gracious letter and for his continued interest in the work of the Fund.

The Speaker seconded the resolution, which was carried by acclamation.

On the motion of the Duke of Norfolk, seconded by Sir Edgar Speyer, it was resolved that the cheques for the grants should be posted on December 20, except in the case of grants for the reservation of beds in consumption sanatoria during 1913, which should be posted on January 2.

### The Executive on the Report on Out-patients.

In the absence of the Earl of Bessborough (Chairman of the Executive Committee), Sir William Collins presented the report of that committee on the subject of the report of the Special Committee on the Out-patient System as follows :—

The Executive Committee have had under consideration during the autumn the report of the Special Committee on the Out-patient System of London which was published and circulated to the hospitals in July last.

The principal recommendations of the Special Committee is the extension of the modern system of almoners already partially in operation at several hospitals. The committee's report describes the work of the almoner, who is an enquiry officer specially trained in the theory and practice both of hospital assistance and of other kinds of charitable or public relief, and whose operations are therefore directed, first, to preventing not only abuse of hospitals, by the method of mere exclusion, but also misuse, by the method of reference to more appropriate medical agencies; and, second, to supplementing the hospital treatment where necessary by means of the co-operation of non-medical agencies.

The Special Committee recognises that the extension of this system to cover the whole ground would involve considerable additional expense, though they mention various factors which might favourably affect this result—notably the probable reduction of the large numbers now attending the out-patient departments, especially should the extension of reform from within be accompanied by a development of provident agencies outside.

The Executive Committee have taken steps to obtain estimates from the various hospitals of the extra cost involved. It is difficult, however, to form an opinion upon the amount of additional work or expense until more is known as to the probable effect of the National Insurance Act generally, and in particular the effect upon the numbers, and character of the ailments, of the out-patients presenting themselves at the hospitals. This factor may also have to be taken into account in connection with the other recommendations of the Special Committee.

The Executive Committee would propose, therefore, to defer for the present making any definite recommendations and to report later. In the meantime they would suggest that the Council should commend the report to the careful consideration of the hospitals concerned and invite their observations and suggestions.

The Future of the Almoner System.

Sir William Collins, in moving the adoption of this report, said that the report did not ask the Council to adopt any principle or rule at the present time. The Report of Lord Mersey stated that the Out-patient Committee in making their recommendation had not considered the possible effect of the National Insurance Act upon the out-patient departments of the London hospitals; and the Executive Committee were of opinion that it would be premature at the present moment to make any recommendation even in regard to the further extension of the almoner system until some greater certainty appeared as to the possible future of the voluntary hospitals as regarded their out-patients' departments under the new scheme of National Insurance. Those acquainted with the administration of the hospitals feared that, notwithstanding the elucidating circulars which had been issued by the Insurance Commissioners and the remarks which they had read in that morning's papers, the voluntary system of hospitals as they had known it in the past would not remain entirely unaffected, at any rate in regard to out-patients, if not also in regard to in-patients, under any National Insurance scheme; the committee thought it premature to make any recommendation and merely asked that this report be received.

The adoption of this report was seconded by Lord Farquhar and carried unanimously.

### The Case of St. George's Hospital.

On the motion of Sir William Collins, seconded by Sir Savile Crossley, the following report of the Executive Committee upon a letter from St. George's Hospital was received :—

"The Executive Committee have had under consideration the following letter addressed to the Fund by the Treasurer of St. George's Hospital, in reply to a request from the Distribution Committee for the information referred to therein :—

'St. George's Hospital,
'November 13, 1912.

'The House Committee have had before them your letter of October 14, 1912, and the subsequent correspondence between the Fund and myself.

'The House Committee desire me to say they are quite willing to place you in possession of the information which they placed before the Lord Mayor when making his investigations on behalf of the Sunday Fund.

'These figures apply to the year 1910, which is the only year for which we have such figures.

'The House Committee also express the wish that the whole circumstances in regard to the suspended grant to St. George's Hospital, to which these figures refer, may be laid before an independent committee, to be agreed upon between the King's Fund and St. George's Hospital.

'Although no resolution was passed on the subject, I think I may take it upon myself to say that, if such a committee were appointed to deal with the whole matter since the suspension of the grant to the hospital, they would be prepared to abide by the decision of such committee of investigation.

'Copies of the information are being printed, and will be sent on to you as soon as possible.

'(Signed) A. William West,
'Treasurer and Chairman.'

"The duty of dealing with the information supplied by St. George's in accordance with this letter rests with the Distribution Committee, since to that committee have been delegated the powers of the General Council with respect to 'the making of inquiries into the circumstances and conditions of . . . . hospitals . . . . applying for grants, and the making of recommendations to the Governors and General Council as to the distribution of the funds of the Corporation to such hospitals . . . . and as to all matters in respect thereof' which powers are further stated to 'extend to ascertaining whether any such hospital . . . . in fact complies with such general rules' as are made by the Fund.

"The Executive Committee are therefore concerned only with that part of the letter which contains the request 'that the whole circumstances in regard to the suspended grant to St. George's Hospital, to which these figures refer may be laid before an independent committee, to be agreed upon between the King's Fund and St. George's Hospital.'

### The Fund's View.

"The Executive Committee have thought it best not to deal with this request themselves, but to lay it before the Council with the following observations :—

"(1) The proposal involves the removal from the Distribution Committee of part of the powers at present delegated to that committee of dealing with the application of an individual hospital.

"(2) The Executive Committee understand that the Distribution Committee are at present engaged on an examination of the fresh evidence submitted by St. George's in support of its renewed application.

"(3) The Executive Committee could not recommend the removal from the Distribution Committee of their powers in respect of an inquiry upon which they are at present engaged; further, they do not consider that the question now raised is one which requires the application of exceptional measures of a kind which do not appear to be contemplated by the Act incorporating the Fund."

Arrangements for 1913.

The Speaker of the House of Commons then said : My Lords and Gentlemen,—We now come to the arrangements for 1913, and I have to announce that the Governors intend to appoint the members of the General Council, Standing Committees, and honorary officers in accordance with the list which I will now ask the Honorary Secretary to read.

The list having been read by Sir Savile Crossley (Hon. Secretary), the Speaker continued :

It now only remains to consider the delegation resolutions which, if approved at this meeting, will only need to be passed formally at the meeting held early in January for that purpose. No alterations are proposed in any of these resolutions, except that the various references to the Out-patient Inquiry Committee are no longer necessary as that committee is *functus officio*.

On the motion of the President of the Royal College of Physicians (Sir Thomas Barlow), seconded by Mr. Sandeman, a resolution was approved delegating powers to the Finance Committee, Distribution Committee, and Convalescent Homes Committee.

On the motion of Sir Cooper Perry, seconded by Mr. Fry, a resolution was approved delegating powers to the Executive Committee.

The Lord Mayor (Sir David Burnett) proposed a vote of thanks to the Speaker of the House of Commons for presiding. The resolution was seconded by the Chairman of the County Council (Lord Cheylesmore) and carried unanimously.

The Speaker having briefly acknowledged the vote of thanks, the proceedings then terminated.

